# A Five-Gene Expression Signature Predicts Clinical Outcome of Ovarian Serous Cystadenocarcinoma

**DOI:** 10.1155/2016/6945304

**Published:** 2016-07-05

**Authors:** Li-Wei Liu, Qiuhao Zhang, Wenna Guo, Kun Qian, Qiang Wang

**Affiliations:** ^1^State Key Laboratory of Pharmaceutical Biotechnology, School of Life Sciences, Nanjing University, Nanjing 210023, China; ^2^School of Life Sciences, Shanghai University, Shanghai 200444, China

## Abstract

Ovarian serous cystadenocarcinoma is a common malignant tumor of female genital organs. Treatment is generally less effective as patients are usually diagnosed in the late stage. Therefore, a well-designed prognostic marker provides valuable data for optimizing therapy. In this study, we analyzed 303 samples of ovarian serous cystadenocarcinoma and the corresponding RNA-seq data. We observed the correlation between gene expression and patients' survival and eventually established a risk assessment model of five factors using Cox proportional hazards regression analysis. We found that the survival time in high-risk patients was significantly shorter than in low-risk patients in both training and testing sets after Kaplan-Meier analysis. The AUROC value was 0.67 when predicting the survival time in testing set, which indicates a relatively high specificity and sensitivity. The results suggest diagnostic and therapeutic applications of our five-gene model for ovarian serous cystadenocarcinoma.

## 1. Introduction

Ovarian serous cystadenocarcinoma is a common female genital cancer that causes more deaths than any other cancer of the female reproductive system. According to Global Cancer Statistics, approximately 230,000 women are diagnosed with ovarian cancer every year, and an estimated 150,000 women die of this disease annually [1]. Ovarian serous cystadenocarcinoma, a type of epithelial ovarian cancer, accounts for about 90% of all ovarian cancers [2]. Studies suggest that the risk factors for the disease include nulliparity, early menarche, late menopause, and family history [3]. Since the disease is often asymptomatic, the majority of patients are diagnosed at an advanced stage, with tumor invasion. Studies showed that the 5-year survival of stage I patients is greater than 90%, while that of patients in stages III to IV is less than 20% [4, 5]. The recent increase in the incidence of ovarian cancer has attracted the interest and attention of researchers worldwide.

With the development of sequencing technology, the research focus has been on the study of signature analysis for prognostic monitoring of ovarian cancer [6–12]. Microarray studies require precise design of probes despite the currently available and well-studied biomarkers for ovarian cancers. Other studies using miRNAs as biomarkers also suggest the limited value for clinical application, and miRNA therapy is still not clinically feasible. Compared with the foregoing methods, gene expression markers not only possess higher practical value, but also yield higher accuracy.

Here, we analyzed 303 clinical samples of ovarian serous cystadenocarcinoma and the corresponding RNA-seq data. We determined the relationship between gene expression data and survival time, in an effort to develop effective and accurate biomarkers for outcome prediction and personalized treatment.

## 2. Materials and Methods

### 2.1. Patient Samples and Gene Expression Data

We collected data from a total of 587 samples of serous cystadenocarcinoma (April 2016) from TCGA (http://cancergenome.nih.gov/) and finally used 303 samples (Table S1, at Supplementary Material available online at http://dx.doi.org/10.1155/2016/6945304) in this study after excluding 284 samples with unknown survival time or insufficient gene expression data. The 303 samples were assigned into 13 batches and randomly allocated to training and testing sets. The prognostic marker model was established with a training set containing 8 batches (batches 9, 11–15, and 17-18) with 168 samples and validated using a testing set, comprising 5 batches (batches 19–22, 24, and 409) with 135 samples.

### 2.2. Statistical Analysis

Initially, we screened the samples by excluding those with unclear survival time or status. We retained only those genes expressed in more than half of the samples for further analysis. The expression level was then determined by logarithmic transformation and univariate Cox regression analysis. The significance of genes with *p* value less than 0.001 was evaluated using random forests. We selected 100 genes of the largest importance to perform multivariate Cox's analysis. Considering the practicality of clinical testing, we established 75,287,520 models with variables ranging from one to five genes using Cox proportional hazards regression analysis [35]. Further, all the 75,287,520 models were subjected to Receiver Operating Characteristic (ROC) analysis and the model with the largest area was selected.

Kaplan-Meier analysis was then conducted in both training and testing groups to validate the efficiency of the model. In order to test the independence and reproducibility of our model, we divided the samples into different datasets according to their ages and disease stages. We then performed Kaplan-Meier analyses and ROC analyses in each condition with IBM SPSS Statistics 22 (http://www.ibm.com/analytics/us/en/technology/spss/).

## 3. Results

### 3.1. Sample Characteristics

According to the screening criteria described, we randomly allocated the 303 samples with explicit survival time, survival state, and expression data into training and testing sets for modeling and validation, respectively. The median age of diagnosis in the selected patients was 58 years, the median survival time was 949 days, and the median survival of late-stage patients was 1069 days. A single patient was found in clinical stage I and 21, 241, and 38 patients were in stages II, III, and IV, respectively. The clinical stages of two patients were unknown (Table 1).

### 3.2. Obtain Genes Associated with Survival Time

Subsequently, we constructed 75,287,520 models comprising factors from 1 to 5 based on the 100 genes with the highest significance in the random forest method. The survival risk score of each patient was calculated according to the corresponding risk formula in each model, and the ROC curves were drawn. We extracted a batch of 5 genes (GPR128, AGXT, CYTH3, C10orf76, and TSPAN9) (Table 2) with the largest AUROC using the following formula: risk score = (0.0796 × expression point of GPR128) + (0.3451 × expression point of AGXT) + (0.3402 × expression point of CYTH3) + (0.6198 × expression point of C10orf76) + (0.2534 × expression point of TSPAN9). All of these genes were reported previously (Table 3). The* CYTH3* gene was expressed in the liver alone, playing a key role in regulating protein sorting and membrane trafficking [21]. Its use as a prognostic molecular marker in liver disease is also discussed. TSPAN9 is probably directly related to the proliferation of cancer cells. Other genes not directly correlated with the development of cancer may affect metabolism via signal transduction and indirectly affect the development of cancer.

### 3.3. Test the Predictive Ability of the Constructed Model Using Testing Set

**Figure 1:**
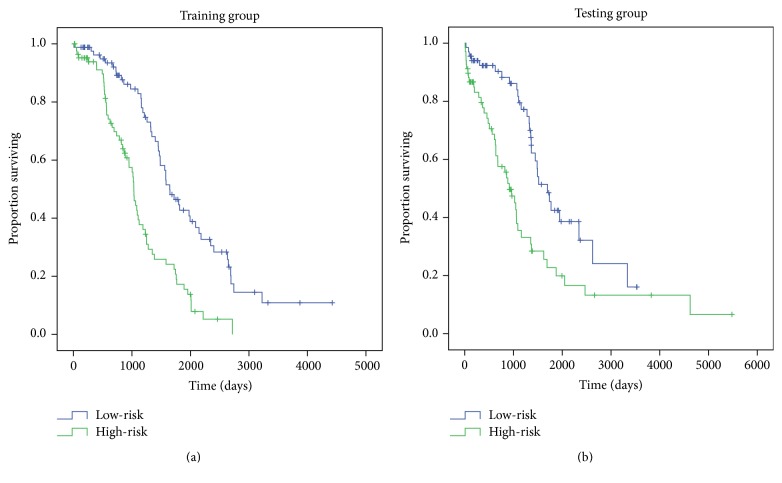
Kaplan-Meier curves with two-sided log rank test show correlation between five-gene model and survival time in both training set and testing set. (a) In training set, by calculating each patient's risk score out of the model, we divided the patients into two groups, named as high-risk group (*n* = 84) and low-risk group (*n* = 84), based on their risk scores. Kaplan-Meier analysis was then performed and significant difference (*p* < 0.001) was found between high-risk and low-risk group in the level of survival time. (b) Similar process and results are showed in testing set.

After constructing the five-variable model with training set, we performed a Kaplan-Meier survival analysis of both training and testing sets to determine its prognostic value. In the training set, by calculating each patient's risk score using the model, we divided the patients into two groups, designated as high-risk (*n* = 84) and low-risk groups (*n* = 84), based on their risk scores. The average survival time of patients in the low-risk group was 1,443 days, longer than in the high-risk group, which was 892 days. Kaplan-Meier analysis indicated a significant difference (*p* < 0.001) between the high-risk and low-risk groups in survival time [Figure 1(a)]. The prognosis of high-risk group appeared worse than that of the low-risk group, indicating that our model successfully distinguished the risk pattern. The higher risk tended to result in shorter survival time. Similar results of Kaplan-Meier analysis were found in the test group [Figure 1(b)], suggesting that our model was universally applicable in determining the risk level and predicting the survival of patients.

**Figure 2:**
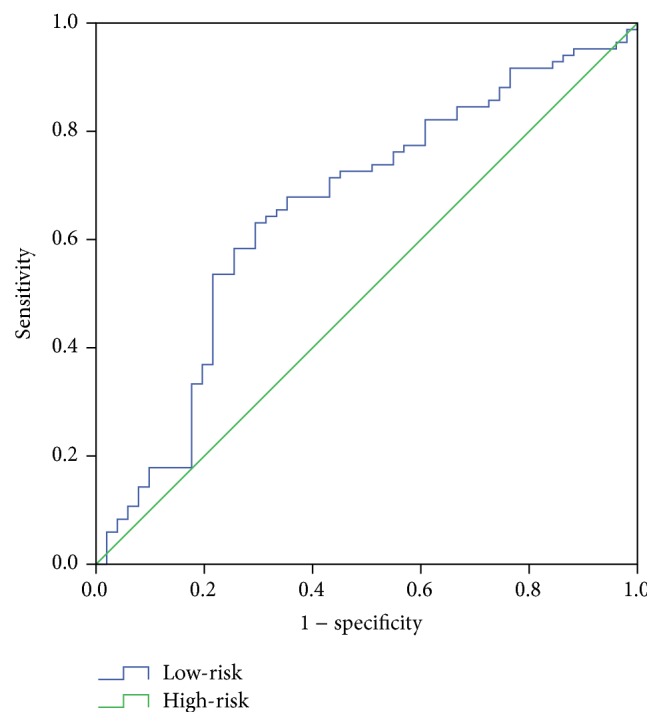
Receiver Operating Characteristic (ROC) analysis of the selected five-gene model. AUROC value is 0.670 (*p* < 0.001).

In order to further confirm the prognostic value of our model in predicting the survival time, we performed ROC analysis of the test group, setting 3 years as the cut-off, and calculated the risk score as the variable. The AUROC value of 0.670 (Figure 2) indicated a relatively high specificity and sensitivity.

### 3.4. The Independence and Reproducibility of the Five-Gene Model

The survival of patients is associated with their age, clinical stage, and other factors. To determine the independence of our model, we conducted a multivariate Cox regression analysis using age and disease stages. We found that the five-gene model was independent of age and disease stage (Table 4).

Further Kaplan-Meier analysis and ROC analysis were then conducted (Table 5). We merged the training and testing sets into an overall dataset, which was divided into two separate groups by age 57. The Kaplan-Meier analysis revealed that, in both groups, patients in low-risk group survived longer than in the high-risk group (*p* ≤ 0.001). Similar results were obtained with the groups of patients at different disease stages (stages I and II were merged because of limited specimen) except stage IV (Figure S1), which may be attributed to the relatively small sample size. However, the AUROC of this group was rather high. These analyses established that our model was independent of other risk factors and successfully distinguished low risk from high risk in each dataset.

## 4. Discussion

Ovarian serous cystadenocarcinoma is a common female genital cancer. Due to the absence of early-stage clinical symptoms and effective diagnosis, most patients were diagnosed with advanced disease. Further, due to the lack of effective treatment, the management of epithelial ovarian cancer is passive. Developing reliable prognostic molecular markers provides meaningful guidance for a reasonable and effective management program.

In this study, we analyzed 303 clinical samples of ovarian serous cystadenocarcinoma and the corresponding RNA-seq data, observed the correlation between gene expression and survival time, and eventually established a risk assessment model based on five factors. Two of these genes (TSPAN9 [30–34], CYTH3 [21–24]) were directly correlated with cancer, with CYTH3 identified as a biomarker in liver cancer.

By calculating each patient's risk score, we found that each set showed significant differences in survival time between low-risk and high-risk groups, indicating that the model accurately predicted the mortality risk. The AUROC value in testing group is 0.670, representing a relatively high specificity and sensitivity.

In conclusion, our gene expression biomarkers can be used for accurate patient risk assessment, demonstrating practical value in predicting clinical outcomes. Our results are based on the samples derived from 303 individuals. Expanding sample size, especially including early-stage cancer patients, will further improve the prognostic value of the model.

## Supplementary Material

Figure S1: Kaplan-Meier curves with two-sided log rank test show correlation between five-gene model and survival time in certain groups. In each set, by calculating each patient's risk score out of the model, we divided the patients into two groups, named as high risk group and low risk group, based on their risk scores. Kaplan-Meier analysis was then performed and significant difference (p<0.001) was found between high risk and low risk group in the level of survival time except in stage IV. (a) patients under the age of 57, (b) patients over the age of 57, (c) patients from stages I and II, (d) patients from stage III, (e) patients from stage IV. Table S1: Clinical sample information of 303 ovarian serous cystuadenocarcinoma patients.

## Figures and Tables

**Table 1:** Assignment of patient demographic and clinical characteristics.

Characteristic	Patients		
	Training set	Testing set	Total
*Age at diagnosis (years)*			
Median	57	59	58
Range	34–87	30–87	30–87
*Vital status*			
Living	58	62	120
Dead	110	73	183
*Follow-up (days)*			
Median	1018	883	949
Median (dead)	1155	919	1069
*Clinical stage*			
Stage I	0	1	1
Stage II	8	13	21
Stage III	142	99	241
Stage IV	18	20	38
Unknown	0	2	2

**Table 2:** Five genes strongly correlated with patients' survival time in training set.

Gene name	*p* value	Hazard ratio	Coefficient	Variable importance	Relative importance
GPR128|84873	0.00092	1.0828	0.0796	0.0009	0.2478
AGXT|189	0.00038	1.4121	0.345	0.0005	0.1442
CYTH3|9265	0.00048	1.4052	0.3402	0.0005	0.1432
C10orf76|79591	0.00037	1.8585	0.6198	0.0009	0.2446
TSPAN9|10867	0.0008	1.2884	0.2534	0.0002	0.0518

**Table 3:** Five-gene functions in previous research.

	Chromosomal	Start site	End site	Function
GPR128	chr3	100328433	100414323	Playing important role in the transduction of intercellular signals across the plasma membrane; related to weight gain and intestinal contraction frequency in mouse [13–16].

AGXT	chr2	240868479	240880502	Expressing proteins involved in glyoxylate detoxification in the peroxisomes; its mutation causes primary hyperoxaluria type I, a severe inborn error of metabolism [17–20].

CYTH3	chr7	6161776	6272644	Mediating the regulation of protein sorting and membrane trafficking; related to HCC (hepatocellular carcinoma) tissues and could serve as prognostic factor [21–24].

C10orf76	chr10	101845599	102056193	Currently unknown; a recent study suggested the loss of C10orf76 resulted in the upregulation of several genes [25–29].

TSPAN9	chr12	3077355	3286564	Mediating signal transduction events that play a role in the regulation of cell development, activation, growth, and motility; associated with adhesion receptors of the integrin family and regulates integrin-dependent cell migration [30–34].

**Table 4:** Cox proportional hazard regression analyses in training and testing sets.

Variables	Univariable model		Multivariable model	
	HR (95% CI)	*p* value	HR (95% CI)	*p* value
*Training group*				
Five-gene model	2.672 (1.801–3.965)	<0.001	2.536 (1.832–3.509)	<0.001
Age	1.683 (1.153–5.457)	0.007	1.013 (0.994–1.031)	0.173
*Testing group*				
Five-gene model	2.248 (1.397–3.620)	0.001	2.224 (1.379–3.586)	0.001
Age	1.224 (0.772–1.941)	0.389	1.153 (0.726–1.830)	0.546

*Training group*				
Five-gene model	2.672 (1.801–3.965)	<0.001	2.725 (1.821–4.078)	<0.001
Stage	1.080 (0.670–1.741)	0.752	0.883 (0.541–1.442)	0.62
*Testing group*				
Five-gene model	2.248 (1.397–3.620)	0.001	2.385 (1.387–3.562)	<0.001
Stage	1.032 (0.580–1.461)	0.453	0.685 (0.432–1.238)	0.428

**Table 5:** Kaplan-Meier analysis and ROC analysis were conducted to validate the reproducibility of five-gene model.

Prognostic factor	Group	Kaplan-Meier *p* value	AUROCs
Age	≤57 (146)	<0.001	0.653
	>57 (157)	0.001	0.683

Stage	I, II (22)	0.018	0.625
	III (241)	<0.001	0.664
	IV (38)	<0.1	0.778
