# Supplementary material for: A Five-Gene Expression Signature Predicts Clinical Outcome of Ovarian Serous Cystadenocarcinoma
Source: Biomed Res Int. 2016 Jul 5;2016:6945304. doi: 10.1155/2016/6945304 (PMC4949334; doi:10.1155/2016/6945304)
Supplement: Supplementary file 1 — Figure S1: Kaplan-Meier curves with two-sided log rank test show correlation between five-gene model and survival time in certain groups. In each set, by calculating each patient's risk score out of the model, we divided the patients into two groups, named as high risk group and low risk group, based on their risk scores. Kaplan-Meier analysis was then performed and significant difference (p<0.001) was found between high risk and low risk group in the level of survival time except in stage IV. (a) patients under the age of 57, (b) patients over the age of 57, (c) patients from stages I and II, (d) patients from stage III, (e) patients from stage IV. Table S1: Clinical sample information of 303 ovarian serous cystuadenocarcinoma patients. [file 6945304.f1.pdf]

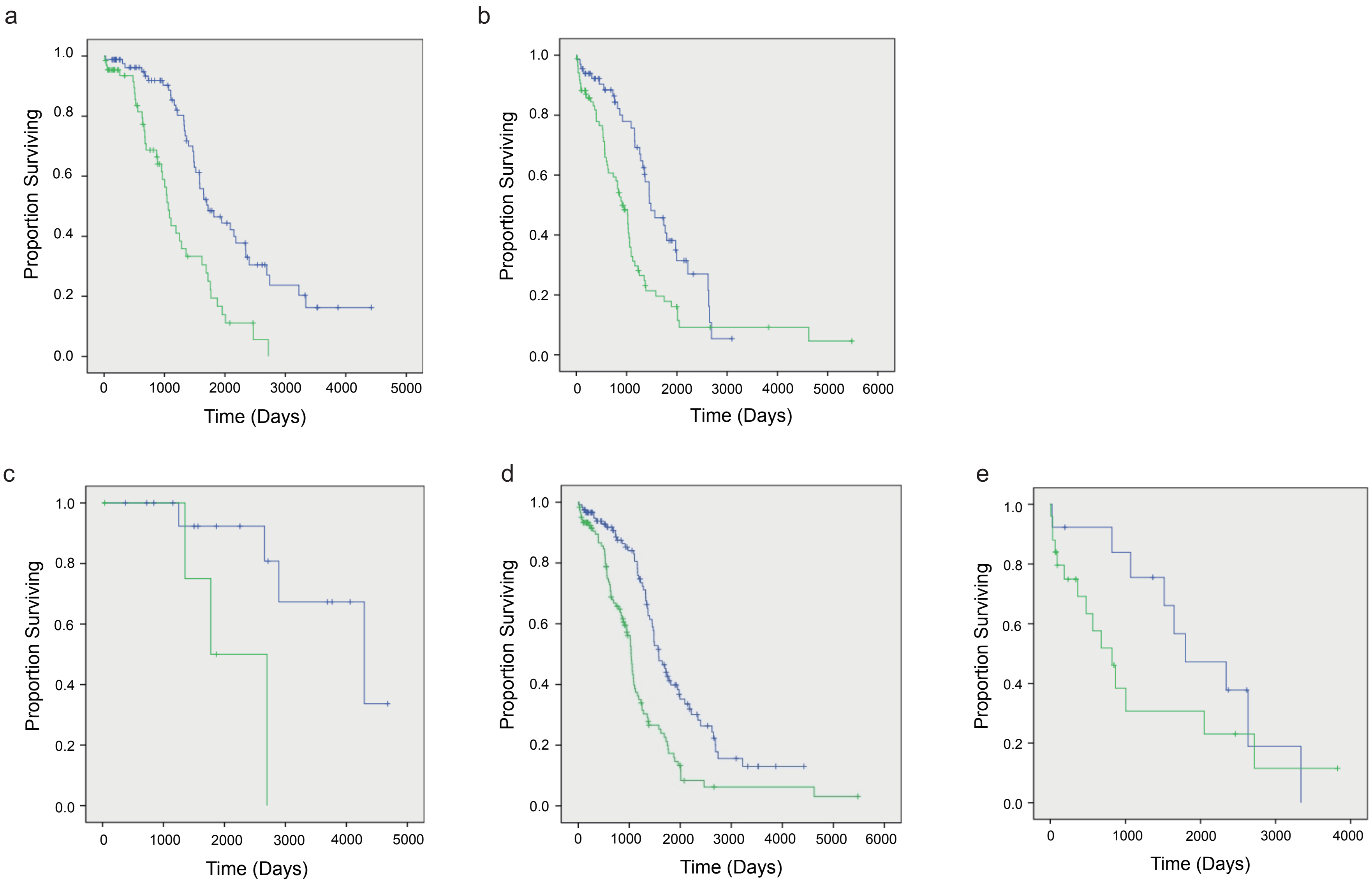

Figure S1 Kaplan-Meier curves with two-sided log rank test show correlation between five-gene model and survival time in certain groups. In each set, by calculating each patient's risk score out of the model, we divided the patients into two groups, named as high risk group and low risk group, based on their risk scores. KAPLAN-MEIER analysis was then performed and significant difference ($p<0.001$) was found between high risk and low risk group in the level of survival time except in stage IV. (a)patients under the age of 57; (b) patients over the age of 57; (c) patients from stages I and II; (d) patients from stage III, (e) patients from stage IV.

Table S1: 303 ovarian serous cystuadenocarcinoma patients' clinical sample information

Sample	Sex	Age	Clinical stage	Vital status	Follow up	Batch
TCGA-04-1348	FEMALE	44	StageIIIB	1	1483	Batch_13
TCGA-04-1362	FEMALE	59	StageIIC	1	1348	Batch_13
TCGA-04-1364	FEMALE	61	StageIIIC	1	1024	Batch_13
TCGA-04-1365	FEMALE	87	StageIIIB	0	2329	Batch_13
TCGA-04-1514	FEMALE	45	StageIIIA	1	1720	Batch_15
TCGA-04-1519	FEMALE	48	StageIIIC	0	24	Batch_17
TCGA-09-0364	FEMALE	80	StageIIC	1	887	Batch_9
TCGA-09-0366	FEMALE	55	StageIIIC	1	1757	Batch_9
TCGA-09-0367	FEMALE	67	StageIIIC	1	547	Batch_9
TCGA-09-0369	FEMALE	56	StageIIIC	1	1082	Batch_9
TCGA-09-1659	FEMALE	51	StageIIIC	1	304	Batch_17
TCGA-09-1662	FEMALE	58	StageIV	1	2717	Batch_17
TCGA-09-1666	FEMALE	57	StageIIIC	0	1752	Batch_17
TCGA-09-1667	FEMALE	61	StageIIC	0	1882	Batch_17
TCGA-09-1668	FEMALE	57	StageIIIC	0	1684	Batch_17
TCGA-09-1669	FEMALE	54	StageIIIA	0	928	Batch_17
TCGA-09-1670	FEMALE	57	StageIIIA	0	547	Batch_18
TCGA-09-1673	FEMALE	50	StageIV	0	92	Batch_18
TCGA-09-1674	FEMALE	79	StageIIIC	0	260	Batch_18
TCGA-10-0928	FEMALE	71	StageIIIC	1	563	Batch_11
TCGA-10-0936	FEMALE	69	StageIIIC	1	1123	Batch_11
TCGA-13-0730	FEMALE	71	StageIIIC	1	542	Batch_9
TCGA-13-0799	FEMALE	44	StageIIIC	0	2535	Batch_9
TCGA-13-0800	FEMALE	52	StageIIIC	0	2661	Batch_9
TCGA-13-0801	FEMALE	46	StageIIIC	1	1399	Batch_9
TCGA-13-0890	FEMALE	56	StageIIIC	0	4424	Batch_11
TCGA-13-0893	FEMALE	48	StageIIIC	1	1319	Batch_13
TCGA-13-0897	FEMALE	54	StageIIIC	1	2182	Batch_11
TCGA-13-0899	FEMALE	60	StageIIIC	1	2012	Batch_11
TCGA-13-0913	FEMALE	53	StageIIIC	0	3325	Batch_11
TCGA-13-0916	FEMALE	49	StageIIIC	0	1785	Batch_11
TCGA-13-0920	FEMALE	65	StageIIIC	1	1484	Batch_11
TCGA-13-0924	FEMALE	45	StageIV	0	2614	Batch_11
TCGA-13-1403	FEMALE	48	StageIIIC	1	2345	Batch_13
TCGA-13-1405	FEMALE	49	StageIV	1	868	Batch_13
TCGA-13-1410	FEMALE	57	StageIV	0	2464	Batch_13
TCGA-13-1411	FEMALE	81	StageIIIC	1	531	Batch_13
TCGA-13-1481	FEMALE	76	StageIIIC	1	2648	Batch_14
TCGA-13-1497	FEMALE	47	StageIIIC	0	3871	Batch_14
TCGA-13-1498	FEMALE	73	StageIIIC	1	2012	Batch_14
TCGA-13-1505	FEMALE	63	StageIIIC	0	1998	Batch_14
TCGA-13-1506	FEMALE	45	StageIIIC	1	1039	Batch_14
TCGA-13-1507	FEMALE	77	StageIIIC	1	1993	Batch_14
TCGA-13-1511	FEMALE	52	StageIV	1	1650	Batch_14

TCGA-13-1512	FEMALE	49	StageIIIC	0	442	Batch_14
TCGA-20-1682	FEMALE	56	StageIIIC	0	837	Batch_18
TCGA-20-1683	FEMALE	65	StageIIIC	0	772	Batch_18
TCGA-20-1684	FEMALE	51	StageIIIC	0	581	Batch_18
TCGA-20-1685	FEMALE	45	StageIIIC	0	508	Batch_18
TCGA-20-1687	FEMALE	46	StageIV	0	81	Batch_18
TCGA-23-1023	FEMALE	65	StageIIIC	0	1233	Batch_12
TCGA-23-1026	FEMALE	45	StageIIIC	0	816	Batch_12
TCGA-23-1027	FEMALE	48	StageIIIC	1	976	Batch_12
TCGA-23-1109	FEMALE	62	StageIIIC	1	1562	Batch_12
TCGA-23-1114	FEMALE	55	StageIIIC	1	2089	Batch_18
TCGA-23-1120	FEMALE	60	StageIIIC	0	130	Batch_12
TCGA-23-1122	FEMALE	53	StageIIIC	1	1189	Batch_12
TCGA-23-1123	FEMALE	59	StageIIIC	1	1018	Batch_12
TCGA-23-1809	FEMALE	63	StageIIC	0	16	Batch_18
TCGA-24-0975	FEMALE	58	StageIIIC	1	663	Batch_12
TCGA-24-1103	FEMALE	50	StageIIIC	1	1646	Batch_12
TCGA-24-1413	FEMALE	51	StageIIIC	0	192	Batch_13
TCGA-24-1416	FEMALE	34	StageIV	0	194	Batch_14
TCGA-24-1417	FEMALE	54	StageIV	0	238	Batch_14
TCGA-24-1418	FEMALE	68	StageIIIC	0	243	Batch_14
TCGA-24-1419	FEMALE	62	StageIIIC	0	239	Batch_14
TCGA-24-1423	FEMALE	61	StageIIIC	0	190	Batch_14
TCGA-24-1424	FEMALE	67	StageIIIC	0	183	Batch_14
TCGA-24-1425	FEMALE	45	StageIIIC	0	181	Batch_15
TCGA-24-1427	FEMALE	58	StageIIIC	0	147	Batch_14
TCGA-24-1428	FEMALE	50	StageIIIC	0	529	Batch_14
TCGA-24-1430	FEMALE	68	StageIIIC	1	863	Batch_14
TCGA-24-1434	FEMALE	59	StageIIIC	1	568	Batch_14
TCGA-24-1435	FEMALE	57	StageIIIC	1	1324	Batch_14
TCGA-24-1436	FEMALE	57	StageIIIC	1	260	Batch_14
TCGA-24-1463	FEMALE	70	StageIIIC	1	2218	Batch_14
TCGA-24-1467	FEMALE	51	StageIIIC	1	3224	Batch_14
TCGA-24-1469	FEMALE	71	StageIIIC	0	277	Batch_15
TCGA-24-1474	FEMALE	57	StageIIIC	1	676	Batch_15
TCGA-24-1544	FEMALE	71	StageIIIC	1	820	Batch_17
TCGA-24-1545	FEMALE	69	StageIIIC	1	1746	Batch_17
TCGA-24-1546	FEMALE	46	StageIIIC	1	1955	Batch_17
TCGA-24-1548	FEMALE	57	StageIIIC	1	493	Batch_17
TCGA-24-1549	FEMALE	58	StageIIIB	1	1721	Batch_15
TCGA-24-1550	FEMALE	49	StageIIIC	1	1249	Batch_15
TCGA-24-1551	FEMALE	53	StageIIIC	1	1579	Batch_15
TCGA-24-1552	FEMALE	77	StageIIIC	1	1259	Batch_15
TCGA-24-1553	FEMALE	53	StageIIIB	1	1767	Batch_15
TCGA-24-1555	FEMALE	50	StageIIIC	1	2692	Batch_15
TCGA-24-1556	FEMALE	50	StageIIB	1	2148	Batch_17
TCGA-24-1557	FEMALE	49	StageIIIC	1	1213	Batch_17
TCGA-24-1558	FEMALE	73	StageIIIC	1	594	Batch_17

TCGA-24-1560	FEMALE	51	StageIIIC	1	1341	Batch_17
TCGA-24-1562	FEMALE	67	StageIIIC	1	1384	Batch_15
TCGA-24-1563	FEMALE	66	StageIIIC	1	1451	Batch_15
TCGA-24-1564	FEMALE	67	StageIIIC	1	787	Batch_15
TCGA-24-1567	FEMALE	54	StageIIIB	1	524	Batch_17
TCGA-24-1603	FEMALE	53	StageIIIB	1	2742	Batch_15
TCGA-24-1604	FEMALE	66	StageIIIC	1	2688	Batch_15
TCGA-24-1616	FEMALE	56	StageIIIC	1	1163	Batch_15
TCGA-24-1847	FEMALE	45	StageIV	0	343	Batch_18
TCGA-25-1312	FEMALE	69	StageIV	1	31	Batch_13
TCGA-25-1313	FEMALE	62	StageIV	1	820	Batch_13
TCGA-25-1314	FEMALE	42	StageIV	1	1004	Batch_13
TCGA-25-1315	FEMALE	50	StageIIIC	1	1583	Batch_13
TCGA-25-1316	FEMALE	55	StageIIIC	1	1279	Batch_13
TCGA-25-1317	FEMALE	66	StageIIIC	1	61	Batch_13
TCGA-25-1318	FEMALE	54	StageIIIC	1	1064	Batch_13
TCGA-25-1319	FEMALE	73	StageIIIC	1	1977	Batch_13
TCGA-25-1320	FEMALE	65	StageIIIC	1	1155	Batch_13
TCGA-25-1321	FEMALE	65	StageIIIC	1	1033	Batch_13
TCGA-25-1322	FEMALE	62	StageIV	1	91	Batch_13
TCGA-25-1323	FEMALE	72	StageIIIC	1	395	Batch_13
TCGA-25-1324	FEMALE	74	StageIIIC	1	1035	Batch_13
TCGA-25-1326	FEMALE	61	StageIIIC	1	1249	Batch_13
TCGA-25-1328	FEMALE	38	StageIIIC	1	2009	Batch_13
TCGA-25-1329	FEMALE	76	StageIIIC	1	457	Batch_13
TCGA-25-1623	FEMALE	71	StageIV	1	565	Batch_17
TCGA-25-1625	FEMALE	66	StageIIIC	1	840	Batch_17
TCGA-25-1626	FEMALE	65	StageIIIC	1	518	Batch_17
TCGA-25-1627	FEMALE	73	StageIIIC	1	394	Batch_17
TCGA-25-1628	FEMALE	67	StageIIIC	1	627	Batch_17
TCGA-25-1630	FEMALE	73	StageIIIC	1	1162	Batch_17
TCGA-25-1631	FEMALE	73	StageIIIC	1	9	Batch_17
TCGA-25-1632	FEMALE	68	StageIV	1	1799	Batch_17
TCGA-25-1633	FEMALE	64	StageIIIC	1	1891	Batch_17
TCGA-25-1634	FEMALE	75	StageIIIC	1	1091	Batch_17
TCGA-25-1635	FEMALE	71	StageIIIC	1	1583	Batch_17
TCGA-29-1688	FEMALE	39	StageIIIC	1	2400	Batch_18
TCGA-29-1690	FEMALE	66	StageIIIC	1	1448	Batch_18
TCGA-29-1691	FEMALE	51	StageIIIC	1	1470	Batch_18
TCGA-29-1693	FEMALE	72	StageIIIC	0	3096	Batch_18
TCGA-29-1694	FEMALE	45	StageIIIC	1	1187	Batch_18
TCGA-29-1695	FEMALE	62	StageIIIC	1	1229	Batch_18
TCGA-29-1696	FEMALE	43	StageIIIC	1	1032	Batch_18
TCGA-29-1697	FEMALE	62	StageIIIC	1	949	Batch_18
TCGA-29-1698	FEMALE	53	StageIIIC	0	2078	Batch_18
TCGA-29-1699	FEMALE	57	StageIIIC	1	1106	Batch_18
TCGA-29-1701	FEMALE	56	StageIIIC	1	515	Batch_18
TCGA-29-1702	FEMALE	84	StageIIIC	1	728	Batch_18

TCGA-29-1703	FEMALE	56	StageIIIC	1	1815	Batch_18
TCGA-29-1705	FEMALE	47	StageIIIC	1	555	Batch_18
TCGA-29-1710	FEMALE	54	StageIIIC	1	951	Batch_18
TCGA-29-1711	FEMALE	45	StageIIIC	0	1053	Batch_18
TCGA-29-1761	FEMALE	80	StageIIIC	1	528	Batch_18
TCGA-29-1762	FEMALE	59	StageIV	1	2634	Batch_18
TCGA-29-1763	FEMALE	43	StageIIC	0	2032	Batch_18
TCGA-29-1770	FEMALE	54	StageIIIC	0	741	Batch_18
TCGA-29-1781	FEMALE	69	StageIIIC	0	255	Batch_18
TCGA-29-1783	FEMALE	58	StageIIIC	0	220	Batch_18
TCGA-29-1784	FEMALE	55	StageIIIC	0	163	Batch_18
TCGA-29-1785	FEMALE	55	StageIIIC	1	1104	Batch_18
TCGA-30-1718	FEMALE	44	StageIIIC	1	1579	Batch_18
TCGA-36-1568	FEMALE	52	StageIIIC	0	875	Batch_17
TCGA-36-1569	FEMALE	52	StageIIIC	0	885	Batch_17
TCGA-36-1570	FEMALE	49	StageIIIC	0	655	Batch_17
TCGA-36-1571	FEMALE	53	StageIIIB	1	695	Batch_17
TCGA-36-1574	FEMALE	48	StageIIIC	0	686	Batch_17
TCGA-36-1575	FEMALE	83	StageIIIB	0	260	Batch_17
TCGA-36-1576	FEMALE	76	StageIIIC	0	915	Batch_17
TCGA-36-1577	FEMALE	43	StageIIC	0	783	Batch_17
TCGA-36-1578	FEMALE	63	StageIV	0	847	Batch_17
TCGA-36-1580	FEMALE	82	StageIIIC	1	737	Batch_17
TCGA-36-1581	FEMALE	63	StageIIC	0	751	Batch_17
TCGA-57-1582	FEMALE	50	StageIIIC	1	731	Batch_17
TCGA-57-1583	FEMALE	57	StageIIIC	1	346	Batch_17
TCGA-57-1584	FEMALE	47	StageIIIC	0	643	Batch_17
TCGA-57-1585	FEMALE	57	StageIIIC	1	53	Batch_17
TCGA-09-2044	FEMALE	77	StageIIB	0	186	Batch_24
TCGA-09-2045	FEMALE	50	StageIV	1	1069	Batch_24
TCGA-09-2048	FEMALE	63	StageIIIC	1	138	Batch_24
TCGA-09-2051	FEMALE	42	StageIIIC	0	1919	Batch_24
TCGA-09-2054	FEMALE	58	StageIIIC	1	637	Batch_22
TCGA-09-2056	FEMALE	62	StageIIIC	0	379	Batch_22
TCGA-13-2060	FEMALE	51	StageIV	0	2369	Batch_24
TCGA-13-A5FT	FEMALE	67	StageIIIC	0	2143	Batch_409
TCGA-23-1029	FEMALE	46	StageIIIC	0	268	Batch_19
TCGA-23-1111	FEMALE	63	StageIIIC	0	98	Batch_19
TCGA-23-2077	FEMALE	45	StageIIIC	0	3525	Batch_22
TCGA-23-2078	FEMALE	66	StageIIIC	0	2661	Batch_22
TCGA-23-2081	FEMALE	49	StageIV	1	2342	Batch_22
TCGA-23-2084	FEMALE	45	StageIV	1	1516	Batch_22
TCGA-24-1842	FEMALE	49	StageIIIC	0	253	Batch_19
TCGA-24-1843	FEMALE	66	StageIIIC	0	106	Batch_19
TCGA-24-1844	FEMALE	64	StageIIIC	0	113	Batch_19
TCGA-24-1846	FEMALE	45	StageIIIC	0	133	Batch_19
TCGA-24-1849	FEMALE	80	StageIIIC	0	176	Batch_19
TCGA-24-1850	FEMALE	72	StageIIIC	0	168	Batch_19

TCGA-24-1924	FEMALE	65	StageIIIC	1	919	Batch_21
TCGA-24-1928	FEMALE	77	StageIIIC	1	336	Batch_21
TCGA-24-1930	FEMALE	53	StageIIIC	1	2467	Batch_21
TCGA-24-2019	FEMALE	46	StageIIIC	0	148	Batch_22
TCGA-24-2020	FEMALE	67	StageIIIC	1	4624	Batch_21
TCGA-24-2023	FEMALE	54	StageIIIA	1	1364	Batch_21
TCGA-24-2024	FEMALE	72	StageIIIC	1	1769	Batch_22
TCGA-24-2026	FEMALE	79	StageIIIC	1	1059	Batch_21
TCGA-24-2027	FEMALE	51	StageIV	1	3337	Batch_21
TCGA-24-2033	FEMALE	87	StageIIIC	1	562	Batch_22
TCGA-24-2035	FEMALE	65	StageIIIC	1	857	Batch_22
TCGA-24-2036	FEMALE	50	StageIIIA	1	1947	Batch_22
TCGA-24-2038	FEMALE	68	StageIIIA	1	1354	Batch_22
TCGA-24-2254	FEMALE	66	StageIIIC	1	1736	Batch_22
TCGA-24-2261	FEMALE	76	StageIIIC	1	24	Batch_22
TCGA-24-2262	FEMALE	57	StageIIIC	1	11	Batch_24
TCGA-24-2267	FEMALE	58	StageIIB	1	1446	Batch_24
TCGA-24-2271	FEMALE	55	StageIIIC	1	962	Batch_24
TCGA-24-2281	FEMALE	68	StageIIA	0	1357	Batch_24
TCGA-24-2288	FEMALE	70	StageIIIC	1	25	Batch_24
TCGA-24-2289	FEMALE	68	StageIV	1	2049	Batch_24
TCGA-24-2290	FEMALE	56	StageIIIC	1	1102	Batch_24
TCGA-24-2293	FEMALE	47	NotAvailable	1	506	Batch_24
TCGA-24-2297	FEMALE	56	StageIIIC	1	1699	Batch_24
TCGA-24-2298	FEMALE	55	StageIIIC	1	1620	Batch_24
TCGA-25-1870	FEMALE	59	StageIIIC	1	455	Batch_21
TCGA-25-1871	FEMALE	70	StageIIIC	1	760	Batch_21
TCGA-25-2042	FEMALE	60	StageIIIC	1	396	Batch_24
TCGA-25-2391	FEMALE	57	StageIIIC	1	1492	Batch_24
TCGA-25-2392	FEMALE	75	StageIV	1	31	Batch_24
TCGA-25-2393	FEMALE	81	StageIIIC	1	1157	Batch_24
TCGA-25-2396	FEMALE	71	StageIIIC	1	92	Batch_24
TCGA-25-2397	FEMALE	59	StageIV	1	365	Batch_24
TCGA-25-2398	FEMALE	71	StageIIIC	1	1369	Batch_24
TCGA-25-2399	FEMALE	80	StageIIIC	1	608	Batch_24
TCGA-25-2400	FEMALE	76	StageIIIC	1	1278	Batch_24
TCGA-25-2401	FEMALE	64	StageIIIC	1	90	Batch_24
TCGA-25-2404	FEMALE	38	StageIIIC	1	883	Batch_24
TCGA-25-2409	FEMALE	71	StageIV	1	821	Batch_24
TCGA-29-1776	FEMALE	63	StageIIIC	0	360	Batch_19
TCGA-29-1778	FEMALE	77	StageIIIC	0	454	Batch_19
TCGA-29-2414	FEMALE	75	StageIIIC	1	2621	Batch_24
TCGA-29-2425	FEMALE	60	StageIIIC	0	1977	Batch_24
TCGA-29-2427	FEMALE	60	StageIIIC	0	1900	Batch_24
TCGA-29-2428	FEMALE	58	StageIIIC	0	1372	Batch_24
TCGA-29-A5NZ	FEMALE	66	StageIIIC	1	1088	Batch_409
TCGA-30-1855	FEMALE	61	StageIIIC	1	75	Batch_19
TCGA-30-1857	FEMALE	64	StageIV	1	8	Batch_19

TCGA-30-1860	FEMALE	58	StageIIIC	1	1366	Batch_21
TCGA-30-1861	FEMALE	74	StageIIIC	1	1058	Batch_21
TCGA-30-1862	FEMALE	65	StageIV	1	186	Batch_21
TCGA-30-1891	FEMALE	61	StageIIIC	1	914	Batch_21
TCGA-31-1944	FEMALE	47	StageIIIC	0	1386	Batch_21
TCGA-31-1946	FEMALE	30	StageIIIC	0	918	Batch_21
TCGA-31-1950	FEMALE	76	StageIIIC	0	571	Batch_21
TCGA-31-1951	FEMALE	58	StageIIIC	0	684	Batch_21
TCGA-31-1953	FEMALE	52	StageIIIC	0	204	Batch_21
TCGA-31-1956	FEMALE	60	StageIIIB	0	1342	Batch_21
TCGA-31-1959	FEMALE	49	StageIV	0	67	Batch_21
TCGA-3P-A9WA	FEMALE	55	StageIIB	0	420	Batch_409
TCGA-57-1993	FEMALE	56	StageIIIC	0	763	Batch_21
TCGA-57-1994	FEMALE	63	NotAvailable	0	761	Batch_21
TCGA-59-2348	FEMALE	59	StageIIIC	0	5481	Batch_24
TCGA-59-2350	FEMALE	44	StageIV	1	679	Batch_24
TCGA-59-2351	FEMALE	51	StageIIIC	0	3532	Batch_24
TCGA-59-2352	FEMALE	78	StageIIIC	1	286	Batch_24
TCGA-59-2354	FEMALE	63	StageIIIC	1	1046	Batch_24
TCGA-59-2355	FEMALE	58	StageIV	1	65	Batch_24
TCGA-59-2363	FEMALE	40	StageIIIA	0	165	Batch_24
TCGA-59-A5PD	FEMALE	55	StageIC	1	624	Batch_409
TCGA-5X-AA5U	FEMALE	61	StageIIC	0	361	Batch_409
TCGA-61-1721	FEMALE	38	StageIV	0	338	Batch_21
TCGA-61-1724	FEMALE	47	StageIIIC	1	637	Batch_21
TCGA-61-1725	FEMALE	40	StageIIIC	0	956	Batch_19
TCGA-61-1728	FEMALE	59	StageIV	0	848	Batch_21
TCGA-61-1733	FEMALE	71	StageIIIC	0	967	Batch_19
TCGA-61-1736	FEMALE	45	StageIIIC	1	1484	Batch_22
TCGA-61-1737	FEMALE	42	StageIV	0	1364	Batch_19
TCGA-61-1738	FEMALE	60	StageIIIC	1	1089	Batch_19
TCGA-61-1740	FEMALE	71	StageIIIC	1	74	Batch_19
TCGA-61-1741	FEMALE	76	StageIIIB	1	1024	Batch_19
TCGA-61-1743	FEMALE	53	StageIIC	1	1329	Batch_21
TCGA-61-1900	FEMALE	51	StageIIIB	0	176	Batch_19
TCGA-61-1907	FEMALE	63	StageIIIC	0	952	Batch_19
TCGA-61-1910	FEMALE	56	StageIIC	0	1127	Batch_19
TCGA-61-1914	FEMALE	65	StageIIIC	0	1722	Batch_19
TCGA-61-1917	FEMALE	60	StageIIIB	1	1321	Batch_21
TCGA-61-1918	FEMALE	45	StageIV	1	479	Batch_21
TCGA-61-1919	FEMALE	58	StageIIIC	1	1161	Batch_21
TCGA-61-1995	FEMALE	43	StageIIIC	0	61	Batch_22
TCGA-61-1998	FEMALE	48	StageIIIC	0	168	Batch_22
TCGA-61-2000	FEMALE	67	StageIIIC	0	441	Batch_22
TCGA-61-2002	FEMALE	46	StageIIIC	0	547	Batch_22
TCGA-61-2003	FEMALE	53	StageIIIC	0	122	Batch_22
TCGA-61-2008	FEMALE	40	StageIIC	0	932	Batch_22
TCGA-61-2009	FEMALE	65	StageIIIC	0	1212	Batch_22

TCGA-61-2012	FEMALE	81	StageIIC	0	932	Batch_22
TCGA-61-2016	FEMALE	51	StageIIIC	1	36	Batch_22
TCGA-61-2088	FEMALE	51	StageIIIC	0	145	Batch_22
TCGA-61-2092	FEMALE	57	StageIIIC	0	1573	Batch_22
TCGA-61-2094	FEMALE	63	StageIIIC	0	2182	Batch_22
TCGA-61-2095	FEMALE	54	StageIIIC	1	1875	Batch_22
TCGA-61-2097	FEMALE	71	StageIIC	0	1844	Batch_22
TCGA-61-2098	FEMALE	62	StageIIIC	0	1993	Batch_22
TCGA-61-2101	FEMALE	55	StageIIIC	1	1688	Batch_22
TCGA-61-2102	FEMALE	74	StageIIIC	1	197	Batch_22
TCGA-61-2104	FEMALE	53	StageIIC	0	2338	Batch_22
TCGA-61-2109	FEMALE	40	StageIIIC	1	629	Batch_22
TCGA-61-2110	FEMALE	56	StageIIIC	1	1354	Batch_22
TCGA-61-2111	FEMALE	61	StageIV	0	3825	Batch_22
TCGA-61-2113	FEMALE	53	StageIIC	1	676	Batch_22
TCGA-OY-A56P	FEMALE	48	StageIIIB	0	1207	Batch_409
TCGA-OY-A56Q	FEMALE	78	StageIIA	0	576	Batch_409
TCGA-VG-A8LO	FEMALE	55	StageIV	1	24	Batch_409
TCGA-WR-A838	FEMALE	72	StageIIIC	1	304	Batch_409
